# Comparison of improvement process of addict and general coma patients hospitalized in Taleghani hospital

**Published:** 2012-11

**Authors:** Mostafa Akbari, Pezhman Soleimani, Farzaneh Kohi, Somayeh Kahrizi

**Affiliations:** ^*a*^Razi University of Kermanshah, Kermanshah, Iran.; ^*b*^Imam Reza Hospital, Kermanshah University of Medical Sciences, Kermanshah, Iran.

## Abstract

**Background:**

Substance and other relative compounds prevents of physiological Adernocorticotropin secretion from pituitary gland and lead to mental exciting and arousal. Present study investigated impact of addiction on improvement process of coma in addiction and general patients.

**Methods:**

170 coma patients hospitalized in Taleghani hospital in 1390 were investigated, that 10 persons of them were addict, therefore 10 addict patients and 10 general patients with GCS 5/6 were selected as control group with access able sampling way. Ti data collection used of patient folders. To data analysis used of T test with spss19.

**Results:**

finding showed that addict patients improvement process is faster than general patients.

Cortizol level in addiction patients is lower than general patients and this lead to mental and physiological arousal in addicts. Therefore to control of problems resulting of substance using incision, kind and dose of drug and substance and harmonic conditions is change. And this mater leads to faster improvement in addictions.

**Conclusions:**

addict patients are dangerous patients. And have to special notes to coma problems and recovery because adrenal gland weakness and default.

**Keywords:**

Addiction, Coma, ICU, GCS 5/6


					Volume 4, Suppl. 1 Nov 2012
Publisher: Kermanshah University of Medical Sciences
URL: http://www.jivresearch.org
Copyright:
Congress of Iranian Neurosurgeons, 14-16 November 2012, Kermanshah, Iran
Paper No. 91
Comparison of improvement process of addict and general
coma patients hospitalized in Taleghani hospital
Mostafa Akbari a,*
, Pezhman Soleimani b
, Farzaneh Kohi a
, Somayeh Kahrizi a
a
Razi University of Kermanshah, Kermanshah, Iran.
b
Imam Reza Hospital, Kermanshah University of Medical Sciences, Kermanshah, Iran.
Abstract:
Background: Substance and other relative compounds prevents of physiological Adernocorticotropin secretion from
pituitary gland and lead to mental exciting and arousal. Present study investigated impact of addiction on
improvement process of coma in addiction and general patients.
Methods: 170 coma patients hospitalized in Taleghani hospital in 1390 were investigated, that 10 persons of them
were addict, therefore 10 addict patients and 10 general patients with GCS 5/6 were selected as control group
with access able sampling way. Ti data collection used of patient folders. To data analysis used of T test with spss19.
Results: finding showed that addict patients improvement process is faster than general patients.
Cortizol level in addiction patients is lower than general patients and this lead to mental and physiological arousal in
addicts. Therefore to control of problems resulting of substance using incision, kind and dose of drug and substance
and harmonic conditions is change. And this mater leads to faster improvement in addictions.
Conclusions: addict patients are dangerous patients. And have to special notes to coma problems and recovery
because adrenal gland weakness and default.
Keywords:
Addiction, Coma, ICU, GCS 5/6
* Corresponding Author at:
Mostafa Akbari: MD, Academic Staff, Razi University of Kermanshah, Kermanshah, Iran. Tell: 0831-8390314, 09188312452,
Email: m.akbari@razi.ac.ir, mostafa457@yahoo.co.uk (Akbari M.).

				

